# Supplementary material for: Highly Porous Hydroxyapatite/Graphene Oxide/Chitosan Beads as an Efficient Adsorbent for Dyes and Heavy Metal Ions Removal
Source: Molecules. 2021 Oct 11;26(20):6127. doi: 10.3390/molecules26206127 (PMC8538019; doi:10.3390/molecules26206127)
Supplement: Supplementary file 1 [file molecules-26-06127-s001.zip › molecules-1409503-supplementary.pdf]

Supplementary Information

Highly porous hydroxyapatite/graphene oxide/chitosan beads as an efficient adsorbent for dyes and heavy metal ions removal

Nguyen Van Hoa[1,*], Nguyen Cong Minh[2], Hoang Ngoc Cuong[3], Pham Anh Dat[1], Pham Viet Nam[4], Pham Hau Thanh Viet[5], Pham Thi Dan Phuong[1], Trang Si Trung[1]

1 Faculty of Food Technology, Nha Trang University, Vietnam; datpa@ntu.edu.vn (PA Dat); danphuong@ntu.edu.vn (PTD Phuong); trungts@ntu.edu.vn (TS Trung)

2 Insitute for Biotechnology and Environment, Nha Trang University, Vietnam; minhnc@ntu.edu.vn (NC Minh)

3 Faculty of Biotechnology, Binh Duong University, Vietnam; hncuong@bdu.edu.vn (HN Cuong)

4 Faculty of Fishery, Ho Chi Minh City University of Food Industry, Vietnam; nampv.fisheries@gmail.com (PV Nam)

5 Faculty of Chemistry, Da Lat University, Vietnam; vietpht@dlu.edu.vn (PHT Viet)

* Correspondence: hoanv@ntu.edu.vn (NV Hoa)

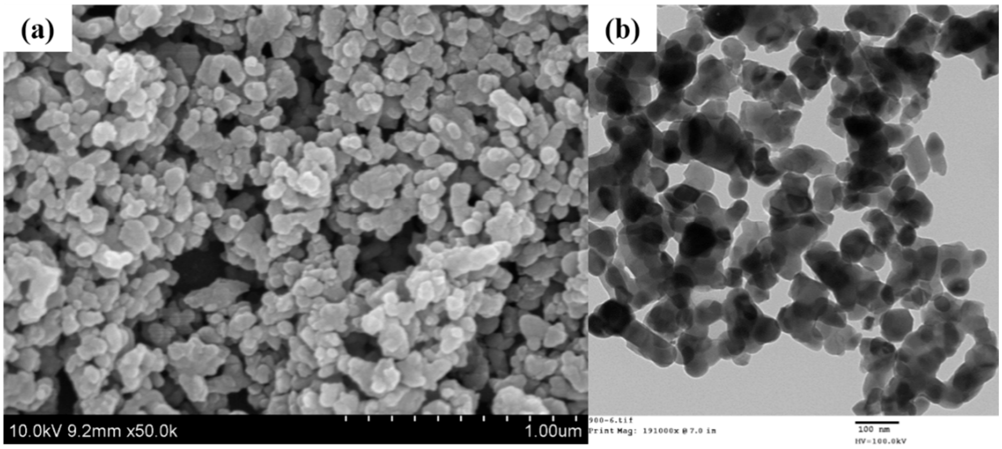

Figure S1. (a) SEM and (b) TEM images of hydroxyapatite.

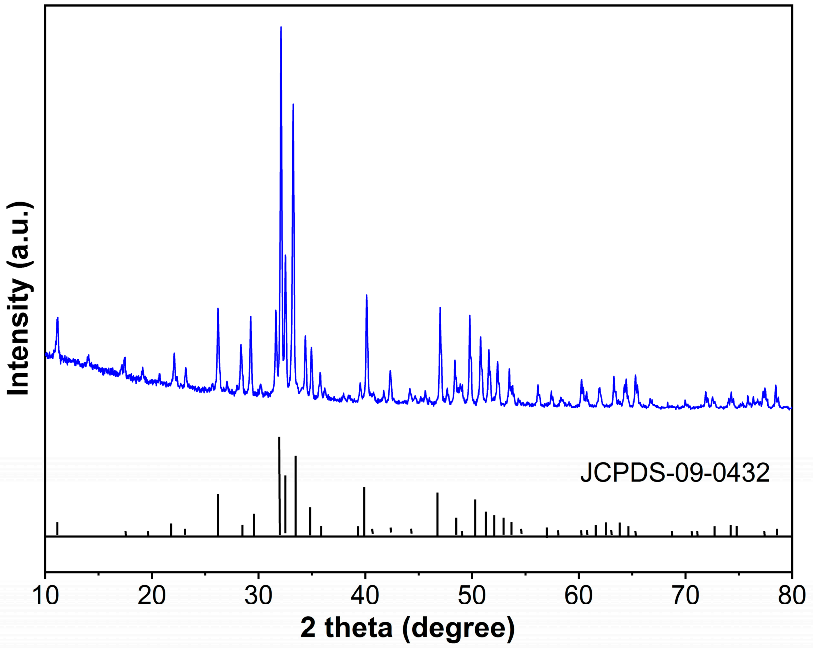

Figure S2. XRD pattern of hydroxyapatite and its JCPDS card No. 09-0432.

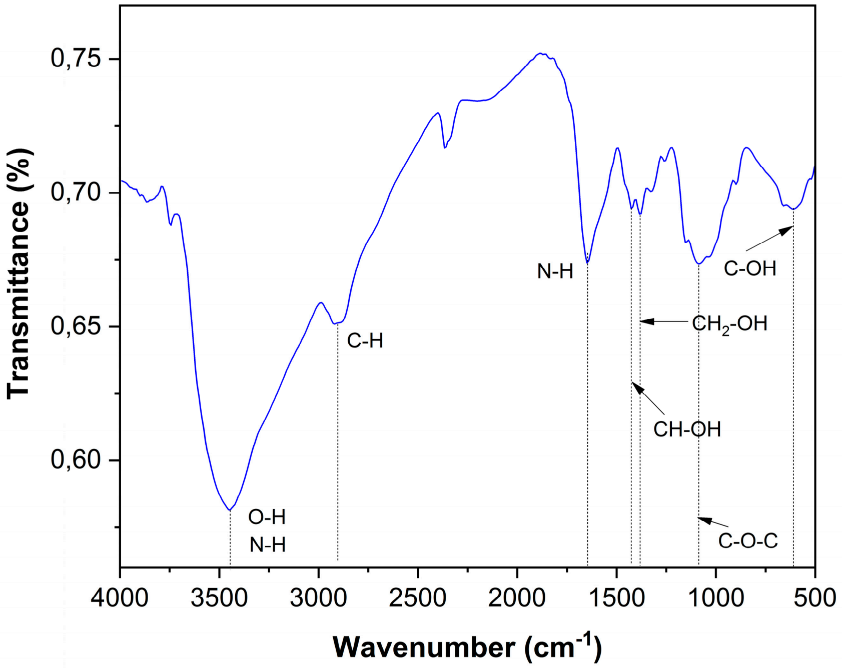

Figure S3. FTIR spectrum of chitosan.

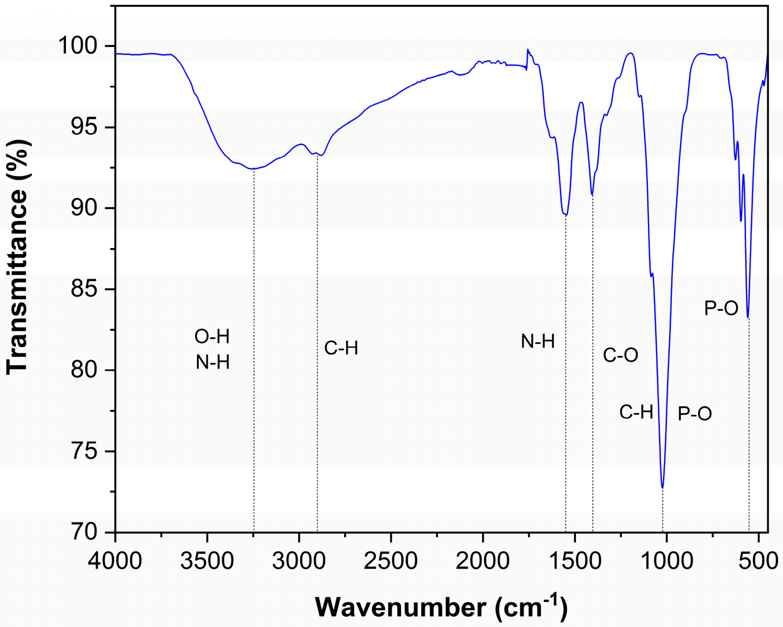

Figure S4. FTIR spectrum of hydroxyapatite.

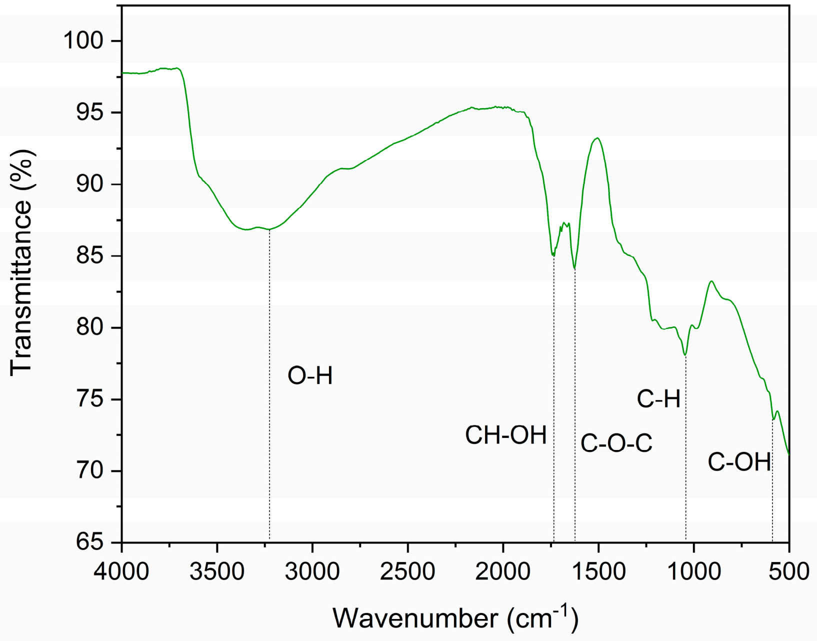

Figure S5. FTIR spectrum of graphene oxide.

Table S1. Maximum adsorption capacity of various chitosan-based materials for MB

Chitosan-based adsorbent	Adsorption capacity (mg g^{-1})	Reference
Cross-linked chitosan/sepiolite composite	40.986	[1]
Lignin-chitosan blends	36.25	[2]
H_2SO_4 crosslinked magnetic chitosan nanocomposite beads	20.408	[3]
Chitosan/Fe_3O_4/GO nanocomposite	30.10	[4]
Chitosan-crosslinked $BiFeO_3$/biochar	18.942	[5]
Chitosan–montmorillonite/polyaniline composite	111.00	[6]
Alginate-chitosanmontmorillonite hydrogel beads	137.2	[7]
Crosslinked chitosan/bentonite composite	97.09	[8]
Chitosan/GO/HA beads	99.00	This study

Table S2. Maximum adsorption capacity of various chitosan-based materials for Cu(II) ions

Chitosan-based adsorbent	Adsorption capacity (mg g^{-1})	Reference
Magnetic chitosan composite	216.6	[9]
Fe_3O_4-chitosan/EDTA composite	225.0	[10]
Malic acid-enhanced chitosan hydrogel beads	183.8	[11]
ZIF-67 modified bacterial cellulose/chitosan composite aerogel	200.6	[12]
Magnetic bentonite/carboxymethyl chitosan/sodium alginate hydrogel beads	56.79	[13]
Chitosan-pectin gel beads	169.4	[14]
Snail shell/hydroxyapatite/chitosan composite	16.741	[15]
Hydroxyapatite-coated-limestone/chitosan composite	130.75	[16]
Chitosan/GO/HA beads	256.41	This study

References

1. Marrakchi, F.; Khanday, W.A.; Asif, M.; Hameed, B.H. Cross-linked chitosan/sepiolite composite for the adsorption of methylene blue and reactive orange 16. *Inter.J. Biol, Macromol.* **2016**, 93, 1231-1239.
2. Albadarin, A.; Collins, M.; Naushad, M.; Shirazian, S.; Walker, G.; Mangwandi, C. Activated lignin-chitosan extruded blends for efficient adsorption of methylene blue. Chem. Eng. J. **2017**, *307*, 264–272.
3. Rahmi; Ismaturrahmi; Mustafa, I. Methylene blue removal from water using H2SO4 crosslinked magnetic chitosan nanocomposite beads. Microchem. J. **2019**, *144*, 397-402.
4. Tran, H.V.; Bui, L.T.; Dinh, T.T.; Le, D.H.; Huynh, C.D.; Trinh, A.X. Graphene oxide/Fe_3O_4/chitosan nanocomposite: a recoverable and recyclable adsorbent for organic dyes removal. Application to methylene blue. *Mater. Res. Express* **2017**, *4*, 035701.
5. Design and preparation of chitosan-crosslinked bismuth ferrite/biochar coupled magnetic material for methylene blue removal. *Int. J. Environ. Res. Public Health* **2020**, *17*, 6-25.
6. Minisy, I.M.; Salahuddin, N.A.; Ayad, M.M. Adsorption of methylene blue onto chitosan–montmorillonite/polyaniline nanocomposite. *Appl. Clay Sci.* **2021,** *203***,** 105993.
7. Wang, W.; Zhao, Y.; Bai, H.; Zhang, T,; Ibarra-Galvan, V.; Song, S. Methylene blue removal from water using the hydrogel beads of poly(vinyl alcohol)-sodium alginate-chitosan-montmorillonite. *Carbohydr. Polym.* **2018**, *198*, 518-528.
8. Bulut, Y.; Karaer, H. Adsorption of methylene blue from aqueous solution by crosslinked chitosan/bentonite composite. *J.Dispers.Sci.Technol.***2015,** 36 ,61-67.
9. Li, J.; Jiang, B.; Liu, Y.; Qiu, C.; Hu, J.; Qian, G.; Guo, W.; Ngo, H.H. Preparation and adsorption properties of magnetic chitosan composite adsorbent for Cu^{2+} removal. *J. Clean. Prod.* **2017**,*158*, 51-58.
10. Chen, B.; Zhao, H.; Chen, S.; Long, F.; Huang, B.; Yang, B.; Pan, X. A magnetically recyclable chitosan composite adsorbent functionalized with EDTA for simultaneous capture of anionic dye and heavy metals in complex wastewater. *Chem. Eng. J.* **2019,** *356,* 69-80.
11. Zhang, Y.; Lin, S.; Qiao, J.; Kołodyńska, D.; Ju, Y.; Zhang, M.; Cai, M.; Deng, D.; Dionysiou, D.D. Malic acid-enhanced chitosan hydrogel beads (mCHBs) for the removal of Cr(VI) and Cu(II) from aqueous solution. *Chem. Eng. J.* **2018,** *353,* 225-236.
12. Li, D.; Tian, X.; Wang, Z.; Guan, Z.; Li, X.; Qiao, H.; Ke, H.; Luo, L.; Wei, Q. Multifunctional adsorbent based on metal-organic framework modified bacterial cellulose/chitosan composite aerogel for high efficient removal of heavy metal ion and organic pollutant. *Chem. Eng. J.* **2020,** *383,* 123-127.
13. Zhang, H.; Omer, A.M.; Hu, Z.; Yang, L.Y.; Ji, C.; Ouyang, X. Fabrication of magnetic bentonite/carboxymethyl chitosan/sodium alginate hydrogel beads for Cu (II) adsorption. *Inter. J. Biol. Macromol.* **2019,** *135,* 490-500.
14. Shao, Z.; Lu, J.; Ding, J.; Fan, F.; Sun, X.; Li, P.; Fang, Y.; Hu, Q. Novel green chitosan-pectin gel beads for the removal of Cu(II), Cd(II), Hg(II) and Pb(II) from aqueous solution. . *Inter. J. Biol. Macromol.* **2021,** *176,* 217-225.
15. Bambaeero, A.; Bazargan-Lari, R. Simultaneous removal of copper and zinc ions by low cost natural snail shell/hydroxyapatite/chitosan composite. *Chin. J. Chem. Eng.* **2021**, *33*, 221-230.
16. Peng, X.; Li, Y.; Liu, S.; Jiang, T.; Chen, W.; Li, D.; Yuan, J.; Xu, F. A Study of adsorption behaviour of Cu(II) on hydroxyapatite-coated-limestone/chitosan composite. *J. Polym. Environ.* **2021**, *29*, 1727-1741.
